# Multitype hand writing as a digital marker for Parkinson's disease

**DOI:** 10.1016/j.prdoa.2026.100480

**Published:** 2026-07-02

**Authors:** Hao Liu, Huishan Deng, Lan Wang, Xinlu Wang, Mingshu Mo

**Affiliations:** a Department of Neurology, the First Affiliated Hospital of Guangzhou Medical University, Guangzhou, No.28, Qiaozhong Road, Guangzhou 510120, China; b Department of Nuclear Medicine, the First Affiliated Hospital of Guangzhou Medical University, Guangzhou, No.28, Qiaozhong Road, Guangzhou 510120, China

**Keywords:** Handwriting, Digital marker, Parkinson's disease, VMAT2, Machine learning

## Abstract

**Background:**

Handwriting impairment is a distinctive symptom of movement disorders (MDs), such as Parkinson's disease (PD) and Parkinsonian syndrome (PDS).

**Objectives:**

Custom-designed digital handwriting (DHW) tools were developed to strengthen the diagnosis of MDs.

**Methods:**

Participants with MDs from the Neurology and PET departments at the First Affiliated Hospital of Guangzhou Medical University were recruited to perform digital handwriting (DHW) tasks, which included drawing a line and a cube, and writing a sentence in Chinese (Ch), English (E), and Korean (K). AV133 PET-CT/MRI was used to detect the expression of vesicular monoamine transporter 2 (VMAT2) in the brain. An attention-based one-dimensional convolutional neural network (1D-CNN) model was developed to evaluate DHW indicators for PD diagnosis.

**Results:**

A total of 197 participants with MDs and 160 matched controls were included, among whom 55 MD patients underwent AV133 PET-CT/MR for VMAT2 quantification. The machine learning model demonstrated that the DHW scores showed excellent discrimination between MDs and controls, with an area under the receiver operating characteristic curve (AUC) of 0.982. In subgroup analyses, the L + E + K and L + E + K + Cu task combinations effectively distinguished PD from PDS, with AUC values of 0.727 and 0.717, respectively. The diagnostic agreement between the DHW and PET-VMAT2 results reached 85.45%. PET-AV133 imaging revealed a significant 33.7% downregulation of VMAT2 expression throughout the putamen in PD patients compared with PDS patients, with dramatic reductions in the posterior putamen. DHW scores from the L + E + K + Cu task combination were significantly correlated with VMAT2 expression in the caudate nucleus (q = 0.0005), specifically within the bilateral caudate heads and bodies.

**Conclusions:**

Custom-designed DHW tasks can serve as effective digital markers for PD diagnosis.

## Highlights

• Applied machine learning to handwriting analysis for movement disorders.
• Developed a designed handwriting task to classify PD.
• Revealed a link between PD-specific neural hardwiring and VMAT2 distribution.

## 1 Introduction

Parkinson's disease (PD) is a neurodegenerative disease characterized by tremors and bradykinesia as common fine motor symptoms and involves the progressive degeneration of the nigrostriatal dopaminergic system [1]. Parkinsonism (PDS), classified as a movement disorder (MD), shares similar motor symptoms with PD but is associated with distinctively impaired brain regions and neurotransmitter disorders [2]. In the course of PD, there is a high risk of fine motor impairments involving handwriting disorders, such as micrographia, a typical feature of the disease [3], [4]. At present, the diagnosis of handwriting disorders relies on the subjective experience of physicians and lacks quantitative standards, making identification in the early stages of PD and PDS difficult [5]. Machine learning may help to explore much more subtle writing details [6].

Handwriting is a representative fine motor skill in humans [7]. The initiation of writing involves language-processing regions such as Wernicke's area for comprehension and Broca's area for expressive planning, along with visual-graphemic and memory-related regions, including the fusiform gyrus, hippocampus, and temporal cortex [7], [8]. The prefrontal cortex subsequently contributes to the structural and stylistic planning of writing [9]. The parietal cortex processes spatial relationships between characters, line spacing, and page layout [10]. The hippocampus and related temporal cortical regions are responsible for retrieving the shapes, writing rules, and grammatical conventions of characters from memory [11]. The motor and supplementary motor cortices decompose the complex movements required for writing and generate precise motor commands [7], [10]. Additionally, writing involves the integration of multiple systems, including the midbrain, which is notably affected in PD, the cerebellum, and the spinal cord, among others [12].

Traditional assessments such as the Montreal Cognitive Assessment (MoCA) include graphic tasks such as cube copying to evaluate cognitive function [13]. Similarly, the DCTclock test uses clock-drawing tasks to assess dementia risk, and other paradigms involve the generation of the drawing of concentric circles [6], [14], [15]. However, these methods focus primarily on static graphic output and overlook dynamic aspects of handwriting, such as writing speed, pressure variation, and spatial trajectory continuity [4]. Other limitations include restricted data quantity, the absence of posture analysis, and insufficient data mining, rendering them inadequate for early PD identification and differentiation from PDS [16], [17].

The vesicular monoamine transporter 2 (VMAT2) is the primary transporter in the central nervous system (CNS) responsible for storing monoamine neurotransmitters, including dopamine and serotonin [18]. VMAT2 is abundantly expressed in the presynaptic terminals of the nigrostriatal pathway, particularly within the striatum [19]. Its dysfunction disrupts dopamine storage and accelerates the degeneration of dopaminergic (DA) neurons in the substantia nigra (SN), a core pathological feature of PD. [20] The ^18^F-AV133 probe selectively binds to VMAT2, and PET imaging with this tracer allows for the objective evaluation of nigrostriatal dopaminergic integrity, aiding in the early diagnosis of PD and its differentiation from PDS [21]. The role of VMAT2 in writing abnormalities remains unclear. In this study, ^18^F-AV133 PET imaging was used to quantify striatal VMAT2 expression. The aim of this study was to investigate the relationship between these findings and detailed handwriting performance, thereby exploring the potential mechanisms underlying handwriting impairments in patients with PD.

## 2 Methodology

### 2.1 Subjects

**Fig. 1:**
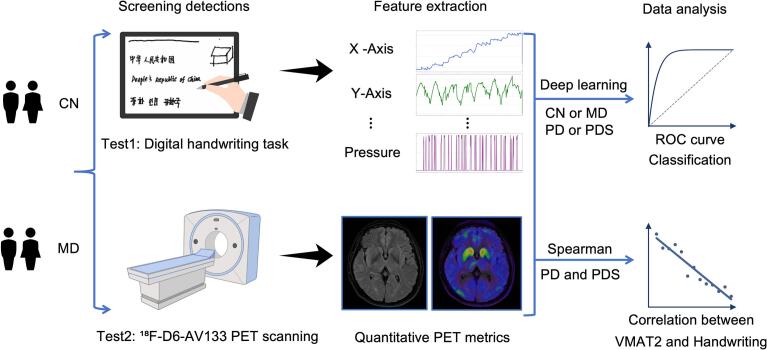
Flow chart of the study design. CN, control; MD, movement disorders; PD, Parkinson's disease; PDS, Parkinsonism; VMAT2, vesicular monoamine transporter 2; PET, positron emission tomography.

A total of 357 Han Chinese subjects, including 197 MDs and 160 matched controls were recruited from the First Affiliated Hospital of Guangzhou Medical University (FAHGMU) from January 2025 to August 2025 as an independent cohort. All diagnoses and neurological assessments were conducted by specialists at the Parkinson's Disease and Related Movement Disorders Unit of the FAHGMU in accordance with the Movement Disorder Society Clinical Diagnostic Criteria for PD. [22] The Unified Parkinson's Disease Rating Scale (UPDRS) and Hoehn and Yahr (H&Y) stages were evaluated by the same designated expert. The participants were required to be native Chinese speakers with the ability to write in Chinese but who were not familiar with Korean or English writing. Those who had recently received anti-PD medications or had a history of cognitive impairment, psychiatric disorders, upper limb motor dysfunction, or an overseas residence exceeding two weeks were excluded. A subset of the recruited participants underwent 18F-AV133 PET imaging to explore the neurobiological mechanisms underlying handwriting impairments. Selection for the PET subgroup was based on voluntary consent and suitability for imaging procedures. Specifically, participants were excluded from PET scanning if they had severe resting tremors that could cause motion artifacts, severe cognitive impairment preventing cooperation during the scan, or contraindications for MRI/CT. These participants were part of the main cohort and were recruited consecutively during the study period, not selected from a separate study (see Fig. 1).

### 2.2 Multitype hand-writing tasks

**Fig. 2:**
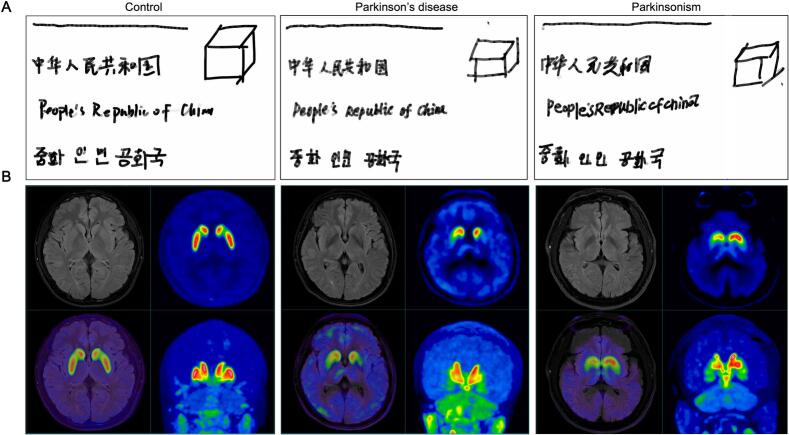
Tasks of digital handwriting and the PET-AV133.

All the writing data were collected through the Wacom Intuos Pro digital tablet and its accompanying Wacom Pro Pen 3, which features a pressure sensitivity of 8192 levels and operates at a 200 Hz sampling frequency. The participants performed a comprehensive multitype handwriting task consisting of 5 subtasks in a predetermined order. These tasks were designed to evaluate fine motor control abilities across different spatial dimensions, including one-dimensional, two-dimensional, and three-dimensional spaces. The tasks included 1) a simple straight-line writing task for one-dimensional motion stability assessment; 2) three text writing tasks involving multiple language systems, namely, the Chinese text of ‘中华人民共和国’, the English text of ‘People's Republic of China’, and the Korean text of ‘중화인민공화국’ for two-dimensional complex motion coordination assessment; 3) a cube drawing task to evaluate three-dimensional visuospatial construction (Fig. 2A). For each writing task, a multidimensional time series data stream was recorded in real time, and the raw signals included the timestamp, x-y coordinates, pen tip pressure, and pen event type. A semiautomatic data processing and feature engineering pipeline was designed to convert the original Wacom signal into a structured feature for machine learning analysis. First, the original data were trimmed of the irrelevant data segments before and after the task based on the first and last valid pen tip pressure signals. Task segmentation was subsequently performed on the writing records using a semiautomated method: an automated algorithm proposed potential task boundaries based on the inflection points of the X-coordinate, after which the researchers manually verified and corrected these boundaries using an interactive visualization tool and labeled the task IDs for each data segment. A special set of dynamic features was extracted from each annotated task segment, including two categories of instantaneous kinematics and dynamic characteristics (IKDC) and behavioral segmentation characteristics (BSC). The IKDC described the core dynamics of pen tip movement, including basic kinematic parameters (horizontal and vertical velocity, acceleration, and jerk), composite kinematic indicators (total velocity, total acceleration), and a series of real-time geometric and dynamic parameters, including displacement increment, cumulative displacement, motion direction angle, trajectory curvature, and pressure change rate. The IKDC can comprehensively quantify the dynamic characteristics of trajectory shape and force control. The BSC refers to behavioral segmentation. The event type of the pen was analyzed, and each writing sequence was divided into the ‘stroke’ stage, which represents the contact between the pen tip and the tablet surface, and the ‘flight’ stage, which represents the movement of the pen tip in the air. A series of macroscopic features for each independent ‘stroke’ and ‘flight’ stage were calculated and included duration, total trajectory length, and horizontal and vertical amplitudes. Additional details are provided in the supplemental data.

### 2.3 PET-AV133 scanning

The method used for type 2 vesicular monoamine transporter (VMAT2) detection by PET-CT/MRI with [^18^F]FP-(+)-DTBZ (AV133) has been reported previously [23]. The PET/CT scanner (Discovery MI, GE Healthcare) was set to a low-dose CT (120 kV, 30 mAs), and brain PET imaging was carried out 70–80 min after the injection of AV133. The image was reconstructed by 3D RAMLA and sharpIR with resolutions of 2.79 × 2.79 × 2.79 mm. PET/MRI was performed by UPMR 790 (3.0 T; United Imaging Healthcare, Shanghai, China). T1-weighted magnetic resonance imaging (T1WI-MRI) sequence parameters included repetition time (TR)/echo time (TE)/inversion time (TI) = 7.9/3/750 ms, 230 mm × 256 mm field of view (FOV), 1 mm section thickness, and a 230 × 256 matrix. PMOD software (version 4.3, PMOD Technologies Ltd., Zurich, Switzerland) was selected to analyze the PET images after normalization to the Hummers-N30R83 template in PMOD using SPM8. The volumes of interest (VOIs) in MRI scans of the brain regions, such as the caudate and putamen, were automatically outlined by the PNEURO tool. The mean score (SUVmean) of the AV133 in the VOIs and the SUV ratio (SUVR) in the target brain region were calculated, and the SUVmean in the occipital cortex was used as the reference (Fig. 2B). Additional details have been provided previously [23].

### 2.4 Machine learning

A two-stage deep learning classification framework was designed. The first stage aimed to distinguish MD patients from healthy controls. In the second stage, PD patients were further distinguished from PDS patients within the identified MD population. The model architecture was designed to integrate a one-dimensional convolutional neural network (1D-CNN) and a task-level attention mechanism (TLAM). The model first processed the input time series data through a feature extraction backbone. For the first-stage classification, the backbone consisted of two convolutional modules, and an additional gated recurrent unit (GRU) layer was added after the convolution module for the second-stage classification. TLAM subsequently weighted and aggregated the features extracted by the backbone network according to the writing task and generated a single context vector. Finally, a classification head received the vector and output the classification probability. Additionally, this classification head could independently evaluate the representations of each task and generate a ‘subtask score’ for each task to quantify the diagnostic contribution of each individual task. This model was trained and evaluated through a rigorous 5-fold stratified cross-validation protocol, which is preferred for datasets of this size to estimate generalization performance more reliably than a single fixed train-test split. In each fold, strict separation between the training and testing sets was maintained to prevent data leakage. The data preprocessing, including quantile cropping and minimum maximum scaling, was fitted solely on the training folds and then applied to the testing fold. In the model optimization, the training set within each fold was further split (90% for training and 10% for internal validation) to monitor the cross-entropy loss and implement the early stopping strategy and dynamic learning rate scheduling. This ensured that the model hyperparameters were tuned without seeing the hold-out test data. The diagnostic performance of this model was evaluated mainly by the area under the receiver operating characteristic (ROC) curve, confusion matrix, and balanced accuracy. The final classification decision was based on the optimal probability threshold that maximized the balanced accuracy on the validation set. Post hoc interpretability analysis of the final model was conducted to understand its decision-making basis. SHapley additive explanations (SHAP) was selected to calculate the contribution of each input feature to the model prediction as the SHAP value and visualize the global feature as a SHAP summary plot.

### 2.5 Statistical analysis

Statistical analyses were performed with SPSS 19.0 (SPSS Inc., Chicago, IL) software and Python (including SciPy, statsmodels, scikit-learn, and Pingouin libraries). The sample size was confirmed using GPower software (version 3.1.9; Heinrich-Heine-Universität Düsseldorf, Düsseldorf, Germany) and justified distinctly for each analysis type. For the machine learning classification tasks, the sample size was determined by the number of eligible participants enrolled during the recruitment period, with model robustness ensured by a rigorous 5-fold cross-validation protocol. For group comparisons, a sample size of 25 participants per group was calculated based on a power of 80% to detect an effect size of Cohen's d = 0.8 at a two-sided significance level of α = 0.05. For correlation analyses, a total sample size of 45 participants was required to detect a correlation of Spearman's |ρ| ≥ 0.40 with 80% power at α = 0.05. Normally distributed data are expressed as the mean ± standard deviation (SD), and other data are expressed as the median (interquartile range). Differences between groups were analyzed using two-way analysis of variance (ANOVA), Mann–Whitney *U* tests, or chi-square tests, depending on the data type and distribution. *p* < 0.05 was considered to indicate statistical significance. For the machine learning component, ROC curve analysis was used to evaluate the ability of the model scores to distinguish diagnostic groups (CN vs. MD; PD vs. PDS). The overall ROC curve was generated by collecting the predicted results of 5-fold cross-validation, and the AUC and 95% confidence interval (95% CI) were calculated. In the group comparisons of written features and PET imaging features, a large-scale derived feature pool from 24 time series signals was constructed and subjected to dimensionality reduction by removing highly collinear features (Spearman correlation coefficient > 0.95 and variance inflation factor > 5.0). The normality of the data was evaluated by the Shapiro–Wilk test to select independent sample *t*-tests or Mann–Whitney *U* tests for intergroup comparisons. For the PET imaging data, a similar statistical testing method was used to compare the SUVR values in various brain regions. To investigate the intrinsic relationship between the DHW score and VMAT2 levels in the combined PET subgroup (PD and PDS), a predetermined stratified correlation analysis was conducted. Partial Spearman rank correlation tests were performed to control for the potential confounding effect of the diagnostic group. This analysis followed a gatekeeper principle to minimize Type I errors: only when the correlation between a higher-level ‘parent’ score and the total striatal uptake value was statistically significant (*p* < 0.05) would its ‘child’ score continue to be tested. In the analyses involving multiple hypothesis testing, *p* values were corrected for the false discovery rate (FDR) using the Benjamini–Hochberg procedure, and a corrected q value<0.05 was considered to indicate statistical significance.

## 3 Results

### 3.1 Digital handwriting score in machine learning-discriminated movement disorders

**Fig. 3:**
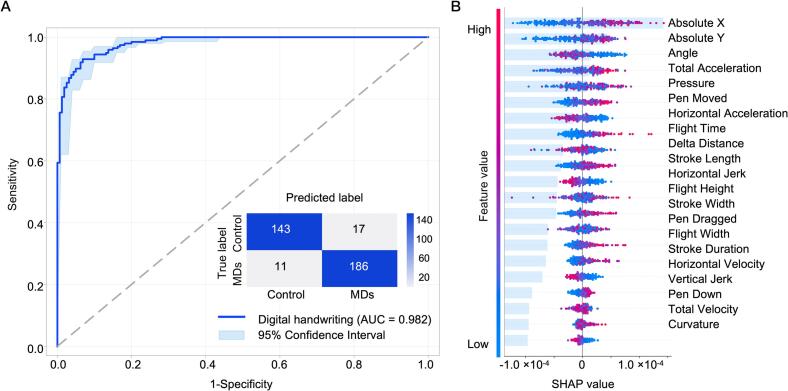
Diagnostic discriminability of the DHW score for movement disorders. A. ROC curves for the effects of the DHW score on movement disorders and the lower-right confusion matrices with predicted and true labels for movement disorder detection; the color scale is shown in blue. B. SHAP values for the DHW score and the impact of each feature ranked by color gradient. MDs, movement disorders; ROC, receiver operating characteristic; AUC, area under the curve; DHW score, digital handwriting score; SHAP, SHapley Additive exPlanations. (For interpretation of the references to color in this figure legend, the reader is referred to the web version of this article.)

The demographic characteristics of the participants with MDs and CNs are provided in Table 1. A total of 197 MD patients and 160 controls were recruited. The MD patients were slightly older than the CN participants but were similar in terms of sex, smoking, and alcohol consumption. The MD group had a short symptom duration with a low UPDRS score and early H&Y stage. The DHW score in the MD group was significantly greater than that in the CN group [97.20 (11.00) vs. 4.99 (18.00), *p* < 0.001; Table 1], indicating strong diagnostic discriminative ability of MDs from CNs, with an AUC of 0.982 (95% CI, 0.9732–0.9901). The confusion matrix revealed a strong performance of the DHW score, with an accuracy of 0.92, a precision of 0.92, and a recall of 0.94 (Fig. 3A). The effects of the top 20 features of DHW on the prediction of the machine learning model are provided in Fig. 3B.

**Table 1:** Summary of sample characteristics.

Variable	Control (*n* = 160)	Movement disorders (*n* = 197)	*p* value
Age (year)	63.00 (19.75)	66.00 (12.00)	0.141 b
Gender (% female)	90 (56.3%)	70 (43.8%)	0.089 a
Smoke (no%)	135 (84.4%)	153 (77.7)	0.110 a
Alcohol (no%)	145 (90.6%)	167 (84.8%)	0.097 a
Disease duration (years)	/	3.0 (5.5)	/
UPDRS	/	42 (33.75)	/
H&Y stage	/	2.0 (1.5)	/
DHW score	4.99 (18.00)	97.20 (11.00)	<0.001 b

UPDRS, Unified Parkinson's Disease Rating Scale, DHW score, digital handwriting score.

a Chi-square test.

b Mann−Whitney test.

**Fig. 4:**
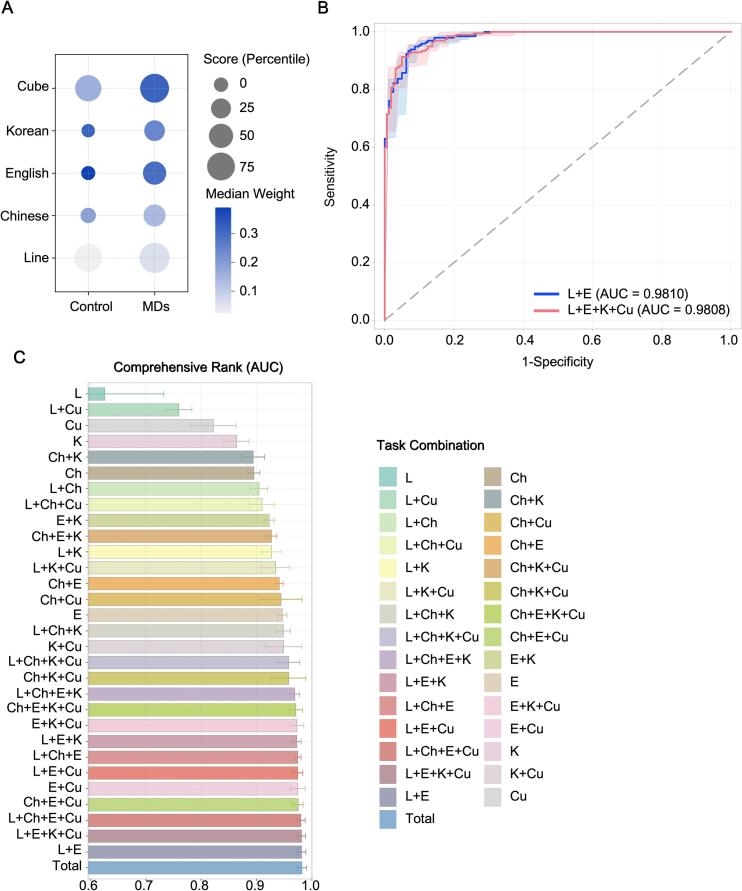
Subgroup analysis of the effect of the DHW score on movement disorders. A. Bubble plot of subgroup writing tasks showing the percentile score and median weight ranked by area and blue color gradient, respectively. B. ROC curves for 1st and 2nd highest multiple-task combination scores on movement disorders. C. Comprehensive ranking of the AUC values for all task combinations with 95% confidence intervals. MDs, movement disorders; ROC, receiver operating characteristic; AUC, area under the curve; L, line; Ch, Chinese; E, English; Cu, cube; K, Korean. (For interpretation of the references to color in this figure legend, the reader is referred to the web version of this article.)

The tasks of the DHW were subclassed into lines, cubes, and sentences of Chinese, English, and Korean. On the basis of the diagnostic scores, the subtasks of cube drawing and sentence writing in Chinese, English, and Korean, as well as the line-drawing task, all showed a significant ability to discriminate MDs from controls (all *p* < 0.001; supplemental data1). In terms of each task's contribution to the model (data shown as median weight), the cube subtask was the most influential feature for identifying MD patients, with the highest median weight within that group (0.2811, p < 0.001 vs. controls; Fig. 4A). The combinations of subtasks in DHW were also analyzed, and the diagnostic discriminability of the other 30 combined types on MDs was determined. The top 2 types of L + E and L + E + K + Cu combinations had AUCs of 0.9810 and 0.9808, respectively, which were similar to that of the total DHW task (Fig. 4B, supplemental data 2).

### 3.2 The digital handwriting score in machine learning discriminated Parkinson's disease from parkinsonism

**Fig. 5:**
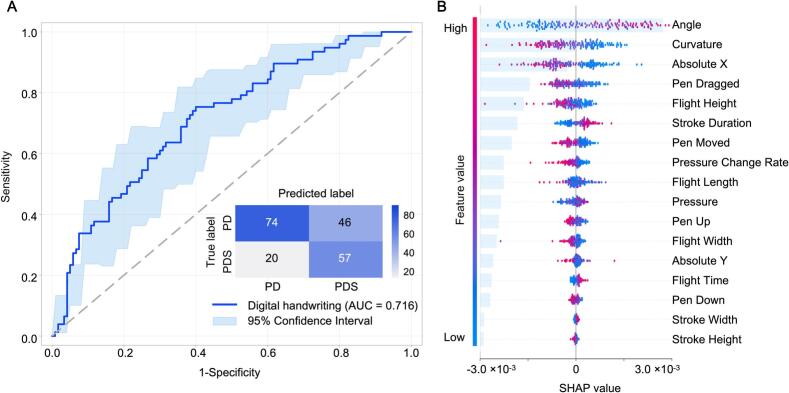
Diagnostic discriminability of the DHW score for Parkinson's disease. A. ROC curves for the DHW score on PD and the lower-right confusion matrices with predicted and true labels for PD discrimination from PDS; the color scale is shown in blue. B. SHAP values for the DHW score and the impact of each feature ranked by color gradient. PD, Parkinson's disease; PDS, Parkinsonism; ROC, receiver operating characteristic; AUC, area under the curve; DHW score, digital handwriting score; SHAP, SHapley Additive exPlanations. (For interpretation of the references to color in this figure legend, the reader is referred to the web version of this article.)

The demographic characteristics of the patients with PD and PDS are provided in Table 2. A total of 120 PD patients and 77 PDS patients were included. Compared with PDS patients, PD patients had similar distributions of age, sex, and H&Y stage; however, they had significantly higher rates of smoking and alcohol consumption, as well as longer disease duration and higher UPDRS scores. In contrast, the DHW score was significantly higher in the PDS group [52.71 (26.00) vs. 42.65 (27.00), *p* < 0.001; Table 2]. Among these patients, 31 PD patients and 24 PDS patients underwent AV133-PET scanning and were assigned to the AV133-PET subgroup. In this subgroup, while most demographic and clinical variables were comparable, the DHW score remained significantly higher in the PDS group [53.25 (29.00) vs. 44.05 (25.00), *p* = 0.002; Table 2]. In the total group, the DHW score had a moderate discriminative ability for differentiating PD from PDS, with an AUC of 0.716 (95% CI, 0.6320–0.7998). In the AV133-PET subgroup, the percentage of diagnostic consistency between DHW and AV133-PET was 85.45% (47/55). The confusion matrix revealed a moderate performance for the DHW score, with an accuracy of 0.66, a precision of 0.79, and a recall of 0.62 (Fig. 5A). The effects of the top 17 features of DHW on the prediction of the machine learning model are provided in Fig. 5B.

**Table 2:** Characteristics of patients with movement disorders.

Variable	AV133 PET Subgroup				Total		
	PD (*n* = 31)	PDS (*n* = 24)	*p* value		PD (*n* = 120)	PDS (*n* = 77)	*p* value
Age (year)	65.00 (17.00)	66 (17.00)	0.420 b		67.00 (10.50)	66.00 (10.55)	0.723 b
Gender (% female)	19 (61.3%)	9 (37.5%)	0.080 a		62 (51.7%)	31 (40.3%)	0.118 a
Smoke (no%)	26 (83.9%)	16 (66.7%)	0.136 a		101 (84.2%)	52 (67.5%)	0.006 a
Alcohol (no%)	28 (90.3%)	17 (70.8%)	0.084 a		107 (89.2%)	60 (77.9%)	0.032 a
Duration (years)	2.00 (2.63)	2.00 (5.00)	0.083 b		3.00 (6.50)	2.00 (3.50)	0.017 b
UPDRS	38.00 (34.00)	34.50 (32.00)	0.218 b		42.00 (40.00)	36.50 (30.25)	0.038 b
H&Y stage	2.00 (2.00)	2.50 (1.00)	0.117 b		2.00 (1.50)	2.00 (1.00)	0.205 b
MoCA	26.00 (7.25)	25.00 (4.00)	0.714 b		22.00 (8.00)	22.50 (9.25)	0.626 b
DHW score	44.05 (25.00)	53.25 (29.00)	0.002 b		42.65 (27.00)	52.71 (26.00)	<0.001 b

PD, Parkinson's disease; PDS, Parkinsonism; UPDRS, Unified Parkinson's Disease Rating Scale; MoCA, Montreal Cognitive Assessment; DHW score, Digital handwriting score.

a Chi-square test.

b Mann−Whitney test.

**Fig. 6:**
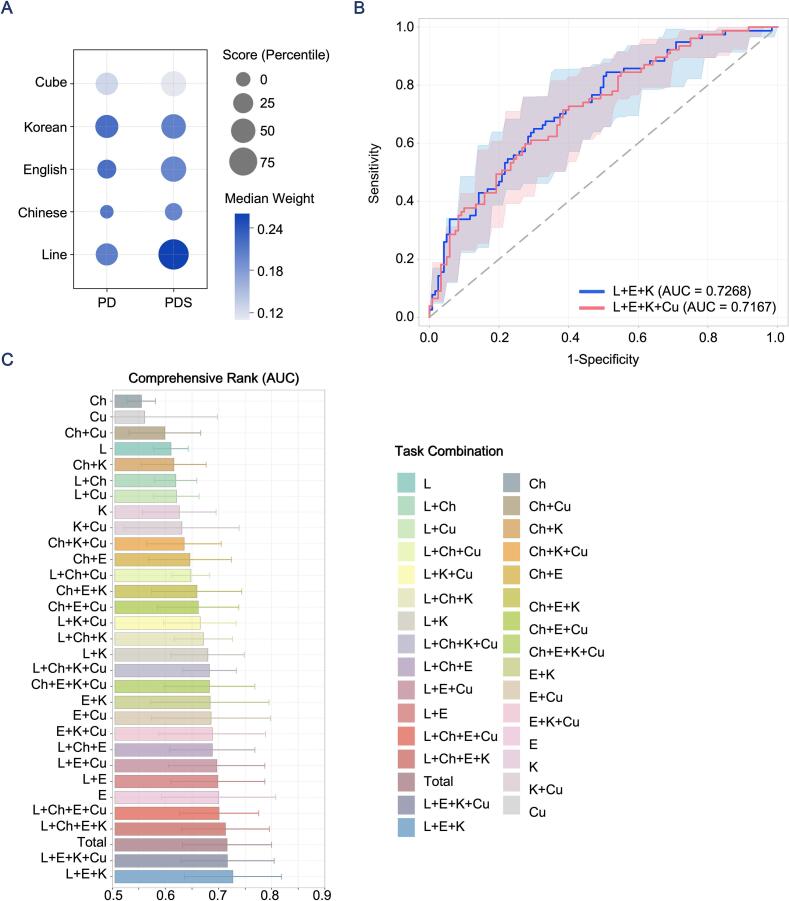
Subgroup analysis of the effect of the DHW score on Parkinson's disease. A. Bubble plot of subgroup writing tasks and the percentile score and median weight ranked by area and blue color gradient, respectively. B. ROC curves for 1st and 2nd multiple-task combination scores for PD discrimination from PDS. C. Comprehensive ranking of the AUC values for all task combinations with 95% confidence intervals. PD, Parkinson's disease; PDS, Parkinsonism; ROC, receiver operating characteristic; AUC, area under the curve; L, line; Ch, Chinese; E, English; Cu, cube; K, Korean. (For interpretation of the references to color in this figure legend, the reader is referred to the web version of this article.)

**Fig. 7:**
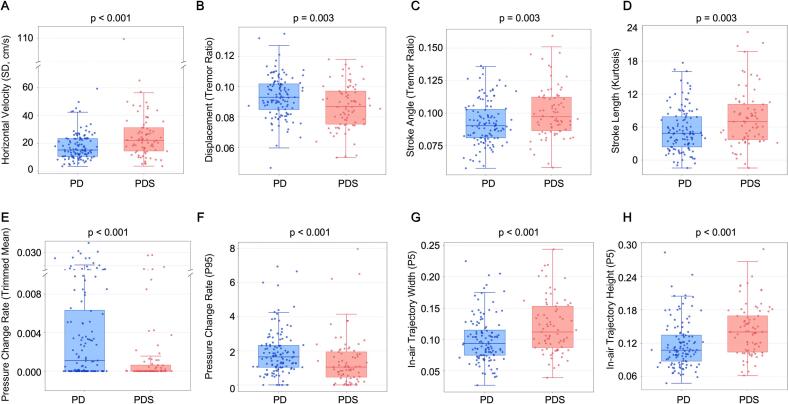
Features of the effect of DHW on PD discrimination.

The single sub-task of line in DHW had the highest absolute values for percentile score and median weight (Fig. 6 A), and its score was significantly different between PD and PDS (*p* = 0.005 in the AV133-PET subgroup and *p* = 0.009 in the total group; supplemental data 3 and 4). The single sub-task of English in DHW had a significantly different score for PD discrimination in the total group (*p* < 0.001), but this significance was not observed in the AV133-PET subgroup (*p* = 0.067). Additionally, the Korean sub-task showed a significant difference in the total group (*p* = 0.003; Supplemental Data 3). The combinations of subtasks in DHW showed different diagnostic discriminability of the other 30 combined types on PD. The top two combinations, L + E + K and L + E + K + Cu, yielded AUCs of 0.727 and 0.717 (Fig. 6B, supplemental data 5), respectively, which were higher than the AUC of the total DHW task (0.716) (Fig. 5A, supplemental data 5). Eight key writing features were identified by machine learning for PD discrimination from PDS, including the standard deviation of horizontal velocity [Horizontal Velocity (SD, cm/s)], the tremor energy ratio derived from displacement [Displacement (Tremor Ratio)), the tremor energy ratio derived from stroke angle [Stroke Angle (Tremor Ratio)), the kurtosis of stroke length [Stroke Length (Kurtosis)), the 10% trimmed mean of the pressure change rate [Pressure Change Rate (Trimmed Mean)), the 95th percentile of the pressure change rate [Pressure Change Rate (P95)), the 5th percentile of the in-air trajectory width [In-air Trajectory Width (P5)), and the 5th percentile of the in-air trajectory height [In-air Trajectory Height (P5)]). The *p*-values for Displacement (Tremor Ratio), Stroke Angle (Tremor Ratio), and Stroke Length (Kurtosis) were 0.003, while those for the other five writing features were < 0.001. All features remained statistically significant after FDR correction (q < 0.05). (Fig. 7).

### 3.3 Correlation between digital hand writing and VMAT2 distribution

**Fig. 8:**
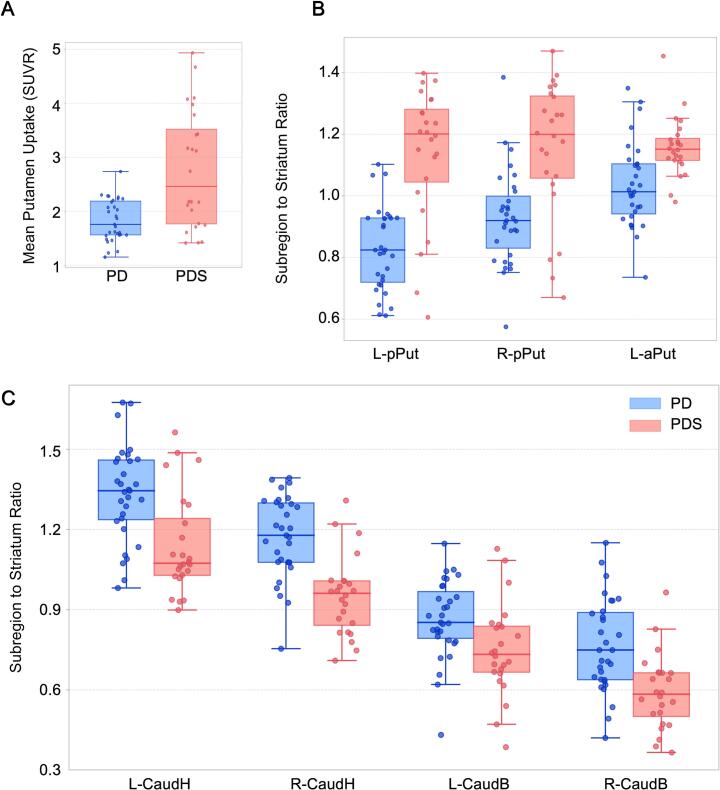
Features of VMAT2 distribution for discriminating PD from PDS.

**Fig. 9:**
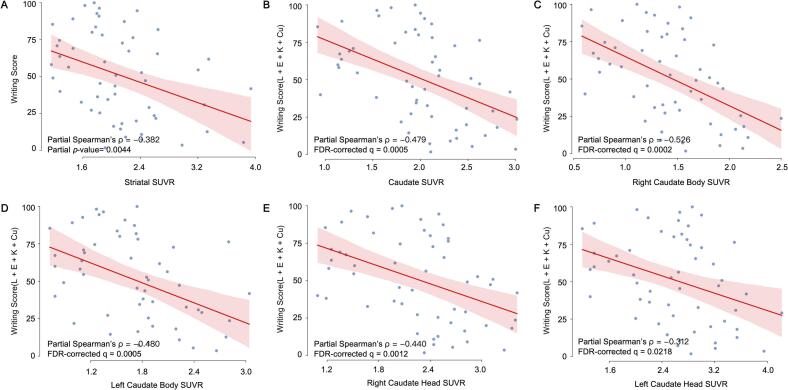
Relationship between multiple-task combinations in the DHW and VMAT2 distribution in the brain. The correlations between multiple-task combinations, namely, the composite score (A), the Line+English+Korean+Cube combination (B—F), and VMAT2 distribution in the striatum and putamen detected by PET-AV133, were analyzed in the PD and PDS by partial Spearman's rank correlation analysis. The significance for Panel (A) is an uncorrected **p** value, while the significance for panels (B—F) is reported as the false discovery rate (FDR)-corrected q value.

In the AV133-PET subgroup, PET-AV133 uptake was lower in the total putamen of PD patients compared with PDS patients (1.83 ± 0.39 vs. 2.76 ± 1.09; q < 0.001; Fig. 8A), corresponding to a 33.7% reduction. This reduction was especially pronounced in the subregions of the striatum, including the L-pPut (0.83 ± 0.14 vs. 1.14 ± 0.22; q < 0.001), R-pPut (0.93 ± 0.15 vs. 1.15 ± 0.22; q < 0.001), and L-aPut (1.03 ± 0.14 vs. 1.16 ± 0.10; q < 0.001; Fig. 8B). In contrast, PD patients showed higher PET-AV133 uptake in the L-CaudH (1.34 ± 0.18 vs. 1.14 ± 0.19; q < 0.001), R-CaudH (1.17 ± 0.15 vs. 0.95 ± 0.15; q < 0.001), L-CaudB (0.86 ± 0.15 vs. 0.75 ± 0.17; q = 0.011), and R-CaudB subregions of the striatum (0.77 ± 0.17 vs. 0.59 ± 0.14; q < 0.001; Fig. 8C). The composite DHW score was significantly negatively correlated with PET-AV133 uptake in the striatum, with a partial Spearman correlation coefficient (ρ) of −0.382 and a partial *p*-value of 0.0044 (Fig. 9A). Analysis of the writing tasks revealed a significant negative correlation between performance on the combination of L + E + K + Cu and PET-AV133 SUVR for the Total Caudate, with a Partial Spearman correlation coefficient (ρ) of −0.479 and an FDR-corrected q-value of 0.0005 (Fig. 9B). Furthermore, the analysis of subregions revealed that this correlation was specifically significant for the Right Caudate Body (ρ = −0.526, q = 0.0002; Fig. 9C), the Left Caudate Body (ρ = −0.480, q = 0.0005; Fig. 9D), the Right Caudate Head (ρ = −0.440, q = 0.0012; Fig. 9E), and the Left Caudate Head (ρ = −0.312, q = 0.0218; Fig. 9F).

## 4 Discussion

### 4.1 Key findings and novel contributions

Current treatments for PD can only alleviate symptoms and do not halt disease progression [24]. Intervention at the early stages of this condition may represent a critical window for stabilizing or even reversing pathological processes in the brain. Impaired fine motor skills are common early motor symptoms in PD patients [24]. We found that machine learning-based analysis of DHW demonstrated strong agreement with AV133-PET imaging for PD and high diagnostic accuracy in differentiating PD patients from healthy controls and PDS patients, as validated by AV133-PET imaging. Through designed writing tasks, a combination of elements (L + E + K + Cu) showed ideal discriminative power for PD identification. AV133-PET results indicated that handwriting abnormalities in PD might be associated with VMAT2 expression in striatum regions. These findings support the potential generalizability of digitally captured handwriting features decoded by machine learning as a diagnostic tool for PD.

### 4.2 Multitype hand writing for early diagnosis of PD

Early diagnosis of PD remains a clinical challenge, and handwriting analysis may help address this issue [25]. Handwriting involves coordinated activity across multiple functional areas of the brain [7]. In early PD, the nigrostriatal system is already impaired [26]. As the disease progresses, the pathology may extend to the parietal, occipital, temporal, and other cortical regions [27]. DHW profiles from early-stage PD patients can more accurately reflect the functional status of the nigrostriatal system [28]. Previous studies have often failed to account for the relationship between disease duration and disease stage, limiting the accuracy and sensitivity of DHW models and reducing their clinical utility [5]. The present study specifically recruited early-stage PD patients, strengthening the association analysis between DHW patterns and nigrostriatal dysfunction. The DHW-based diagnostic model showed 85.45% consistency with the AV133-PET findings, demonstrating an accuracy of 0.92 and a sensitivity of 0.94 in distinguishing MDs from healthy controls. Compared with subjective evaluation, machine learning enables in-depth mining of subtle DHW features [15], [29]. An interpretability analysis using SHAP was performed to understand the model's decision-making process. Analysis of the top 20 features (Fig. 3B) revealed that the Absolute X, Absolute Y, and Angle were the three most influential characteristics for discriminating MDs from controls. These objectively quantified features provide detailed insight into fine motor impairments in PD patients.

Both PD and PDS patients may exhibit dysgraphia, which is difficult to differentiate in early disease stages [30]. Traditional handwriting analysis relies largely on qualitative observation, whereas machine learning-assisted quantitative evaluation may offer more precise diagnostic markers [30]. Unlike PD, where pathology is predominantly centered in the SN, PDS often involves more widespread brain regions [31]. For example, multiple system atrophy (MSA), a subtype of PDS, is frequently accompanied by atrophy in the autonomic, cerebellar, and pyramidal systems, in addition to PD-like motor impairments [32]. Similarly, progressive supranuclear palsy (PSP), another PDS subtype, is characterized by midbrain atrophy, ocular movement deficits, and neuropsychiatric symptoms [33]. Owing to divergent neuroanatomical involvement, PDS patients exhibit handwriting profiles distinct from those of PD patients. MSA patients often display irregular tremor and ataxia-related handwriting abnormalities beyond micrographia, whereas PSP patients show signs of macrographia and spatial misalignment due to visuospatial and oculomotor dysfunction [34]. Currently, differentiation relies on subjective assessment and lacks quantitative standards. To interpret the model's ability to discriminate PD from PDS, a SHAP analysis was conducted. Among the top 17 features visualized (Fig. 5B), Angle, Curvature and Absolute X were revealed to be the most informative for model classification. The resulting model achieved an accuracy of 0.66, a precision of 0.79, and a sensitivity of 0.62. These findings contribute to a more accurate digital handwriting profile for PD and offer a quantitative basis for its differential diagnosis against PDS.

It is notable that the AUC for discriminating PD from PDS was substantially lower than that for discriminating MDs from controls, a discrepancy that can be understood from several perspectives. First, the MD-versus-control classification is fundamentally a distinction between a pathological state and a normal state, whereas the PD-versus-PDS classification involves differentiating between two conditions that both affect the basal ganglia-thalamocortical motor circuit, resulting in inherent overlap in kinematic features such as velocity, pressure, and trajectory patterns [2]. Second, the PDS group comprises multiple clinical subtypes, including MSA, PSP, and CBD, each with distinct handwriting impairment profiles [32], [33], [34], [35]. Pooling these heterogeneous subtypes into a single PDS category inevitably blurs the classification boundary with PD. Third, the early-stage differential diagnosis between PD and PDS is a well-recognized clinical challenge, even for experienced movement disorder specialists [2]; the combination of well-designed DHW tasks and AV133-PET may help to slightly reduce this inherent difficulty in this study. Future studies may improve the specificity of DHW-based differentiation in the following directions: expanding the sample size of individual PDS subtypes; introducing additional writing task dimensions to capture a richer spectrum of motor features; integrating DHW with imaging or blood-based biomarkers; and conducting longitudinal follow-up studies.

The design of handwriting tasks plays a key role in the identification of CNS diseases [5]. Compared with tasks focused on motor disorders, drawing tasks are more closely associated with cognitive impairment [35]. Drawing requires the coordination of multiple brain regions and involves cognitive functions such as memory, executive function, visuospatial processing, motor planning, and abstract thinking [36]. Patients with AD who exhibit drawing difficulties often show dysfunction related to spatial structure and compositional organization, whereas those with FTD may display impairments in executive function and planning that affect the drawing process [37]. Such abnormalities are seldom observed in early-stage PD patients but may appear in advanced PD patients and some PDS patients. To enhance the diagnostic value of DHW, in this study, we designed a three-dimensional DHW task supplemented with drawing content. The one-dimensional subtask of straight-line drawing aimed to evaluate coordinated CNS function. The two-dimensional subtask involved character writing—including English (an alphabetic language) as well as Chinese and Korean (ideographic languages)—to engage distinct motor control mechanisms [38]. In accordance with the participant inclusion criteria, Chinese writing was considered a familiar native language, whereas English and Korean were treated as unfamiliar foreign languages. This distinction facilitated the comparison between highly automated motor skills and novel motor planning. Additionally, aspects such as font structure and text layout (e.g., word and sentence arrangement) are largely influenced by fine motor control and coordination regulated by the midbrain and cerebellum [39], [40]. The three-dimensional subtask of cube drawing was used to evaluate the parietal cortex, hippocampus, and their collaborative networks [41]. The combination of handwriting subtasks was evaluated, revealing that the L + E + K + Cu and L + E + K combinations had high diagnostic value for PD. Interestingly, the exclusion of the native Chinese task improved discriminative performance. We postulate that writing in a native language is a highly over-learned and automated process that may be preserved in early PD through compensatory cortical mechanisms. In contrast, copying unfamiliar characters, as a novel motor planning task, requires intense executive control and visuospatial integration. This imposes a substantial cognitive and motor load on the compromised basal ganglia-thalamocortical loops, making deficits in these novel tasks more pronounced and offering superior discriminative value [42]. Further evidence is needed to confirm these observations. In conclusion, this study underscores the importance of the tailored DHW task design in enhancing early PD diagnosis.

VMAT2 may indirectly influence handwriting-related neural circuits by regulating monoamine neurotransmitter systems in the brain [19]. Handwriting is a complex fine motor task that relies on the dopamine-mediated basal ganglia–thalamocortical motor loop [43]. It also involves cognitive processes such as attention, planning, and emotional states [7]. VMAT2 contributes to the regulation of norepinephrine—produced in the locus coeruleus—which modulates attention, alertness, and arousal [19]. It also affects serotonin pathways, which may influence mood and thereby impact handwriting [44]. In the brain, VMAT2 transports dopamine, norepinephrine, serotonin, and other monoamine neurotransmitters into synaptic vesicles, protecting them from cytoplasmic degradation [44]. In this study, AV-133 was used to examine the relationship between DHW impairments and VMAT2 expression [23]. Group comparisons confirmed the typical pattern of dopaminergic loss in early PD, with severe deficits observed in the posterior putamen. Interestingly, the DHW abnormalities from the L + E + K + Cu task combination did not correlate most strongly with this area of peak pathology. Instead, they showed significant correlations with VMAT2 expression in caudate nucleus, specifically involving the bilateral caudate bodies and heads. The caudate nucleus is a key component of the associative striatum, playing a crucial role in motor planning, cognitive flexibility, and the learning of new motor sequences [45]. This distribution suggests that while the sensorimotor striatum (posterior putamen) is severely depleted in early PD, the execution of our novel, cognitively demanding handwriting tasks (e.g., unfamiliar languages and cube drawing) relies heavily on the remaining function of the associative circuits. Consequently, the observed handwriting impairments likely reflect the functional limit of these compensatory associative networks driven by dopamine levels in the caudate. This hypothesis warrants further investigation.

### 4.3 Limitations

Some limitations should be noted. First, our PET analysis did not perform partial volume correction (PVC) [46]. Compared with early-stage PD, striatal atrophy is more common in PDS such as MSA and PSP, which may exacerbate partial volume effects (PVE) and lead to underestimation of radiotracer concentration independent of actual VMAT2 density loss [47]. However, from a clinical diagnostic perspective, this atrophy-induced signal reduction effectively acts as a ‘beneficial artifact’, particularly in the caudate region where PD patients typically retain relatively preserved uptake. The superimposed atrophic effect in PDS further reduces signal intensity in these regions, thereby amplifying the contrast in striatal gradients between PD and PDS [48]. The PDS patients generally exhibit both VMAT2 loss and structural atrophy, uncorrected SUVR captures the specific combined impact of these pathological changes, potentially enhancing the discriminant sensitivity of this diagnostic model [49], [50] Second, although our model achieved high diagnostic performance, this remains a single-center validation study. While we employed a rigorous 5-fold cross-validation scheme to estimate generalization error, future multicenter validation in larger and more diverse cohorts is necessary to further confirm the robustness and clinical utility of the DHW score. Third, all handwriting data were acquired using a professional-grade digital tablet (Wacom Intuos Pro), and this hardware dependence may limit accessibility in primary care and home-monitoring settings. Future work should explore adaptation to consumer-grade touch-screen devices, which would require re-validation of feature extraction fidelity and diagnostic performance.

## 5 Conclusions

Handwriting serves as a valuable biomarker for the early diagnosis of PD. The tailored design of handwriting tasks significantly enhances their diagnostic utility for CNS disorders. Among these tasks, the L + E + K + Cu combined task demonstrates strong potential as an auxiliary tool for diagnosing PD. Handwriting impairments observed in this task are closely associated with reduced VMAT2 expression in the caudate nucleus. These findings suggest that dopamine-dependent neural circuits involving the caudate, along with cortical areas such as the angular and supramarginal gyri, may modulate PD-related handwriting deficits.

Boxplots display the distribution of eight key writing features for PD discrimination from PDS. The writing features were identified by machine learning with a two-step collinearity filter (based on correlation and variance inflation factor, VIF) and a subsequent correction for multiple comparisons using the false discovery rate (FDR) method. (A) The standard deviation of horizontal velocity [Horizontal Velocity (SD, cm/s)]. (B) The tremor energy ratio derived from displacement [Displacement (Tremor Ratio)]. (C) The tremor energy ratio derived from stroke angle [Stroke Angle (Tremor Ratio)]. (D) The kurtosis of stroke length [Stroke Length (Kurtosis)]. (E) The 10% trimmed mean of the pressure change rate [Pressure Change Rate (Trimmed Mean)]. (F) The 95th percentile of the pressure change rate [Pressure Change Rate (P95)]. (G) The 5th percentile of the in-air trajectory width [In-air Trajectory Width (P5)]. (H) The 5th percentile of the in-air trajectory height [In-air Trajectory Height (P5)]. The *p*-value displayed in each panel represents the uncorrected p-value derived from an independent statistical test (either a Mann-Whitney *U* test or an independent *t*-test, depending on data normality). All features remained statistically significant after FDR correction (q < 0.05).

Boxplots display the distribution of VMAT2 in the brain for discriminating PD from PDS. (A) Expression of VMAT2 for the total putamen; the subregions of the striatum included L-pPut, R-pPut, and L-aPut (B), and the subregions of the caudate nucleus included L-CaudH, R-CaudH, L-CaudB, and R-CaudB (C). L-pPut, Left Posterior Putamen; R-pPut, Right Posterior Putamen; L-aPut, Left Anterior Putamen; L-CaudH, Left Caudate Head; R-CaudH, Right Caudate Head; L-CaudB, Left Caudate Body; R-CaudB, Right Caudate Body.

## CRediT authorship contribution statement

**Hao Liu:** Software, Project administration, Methodology, Formal analysis, Data curation. **Huishan Deng:** Writing – original draft, Visualization, Validation, Resources, Project administration, Methodology, Data curation. **Lan Wang:** Visualization, Validation, Resources, Methodology, Formal analysis, Data curation. **Xinlu Wang:** Validation, Software, Resources, Methodology, Data curation. **Mingshu Mo:** Writing – original draft, Supervision, Investigation, Funding acquisition, Conceptualization.

## Consent to publish

Patients provided written consent for publication.

## Ethical approval and consent to participate

This study was conducted in accordance with the World Medical Association's Declaration of Helsinki and all subsequent amendments. The study protocol was approved by the Institutional Review Board of FAHGMU (ID: ES-2025-142-01) at January 2025. Patients provided written consent to participate in this investigation.

## Funding

This work was supported by 10.13039/501100001809the National Natural Science Foundation of China (81701254).

## Declaration of competing interest

The authors declare that they have no known competing financial interests or personal relationships that could have appeared to influence the work reported in this paper.

## Data Availability

Data will be made available by the corresponding author upon request. All baseline imaging and cognitive data are available to the research community upon request at https://github.com/mishmoth/Multitype-hand-writing-as-a-digital-marker-for-Parkinson-s-disease- The underlying code for this study is not publicly available but may be made available to qualified researchers on reasonable request from the corresponding author.
