# Supplementary material for: Multitype hand writing as a digital marker for Parkinson's disease
Source: Clin Park Relat Disord. 2026 Jul 2;15:100480. doi: 10.1016/j.prdoa.2026.100480 (PMC13377146; doi:10.1016/j.prdoa.2026.100480)
Supplement: Appendix A — Supplementary material 1 [file mmc1.docx]

**Supplemental data 1****. Subtask analysis** **of** **movement disorders**

| Variable | Percentile score | | | Median Weight | | |
| --- | --- | --- | --- | --- | --- | --- |
|  | Control (n =160) | Movement disorders (n =197) | *p*value | Control (n =160) | Movement disorders (n =197) | *p*value |
| Line | 99.89 (87.00) | 99.99 (3.00) | <0.001^a^ | 0.0066 (0.05) | 0.0386 (0.10) | <0.001^a^ |
| Chinese | 1.99 (12.00) | 68.00 (61.00) | <0.001^a^ | 0.1569 (0.07) | 0.1132 (0.09) | <0.001^a^ |
| English | 1.10 (9.00) | 85.81 (48.00) | <0.001^a^ | 0.3458 (0.11) | 0.2708 (0.19) | <0.001^a^ |
| Korean | 0.72 (8.00) | 39.16 (62.00) | <0.001^a^ | 0.2813 (0.10) | 0.2168 (0.11) | <0.001^a^ |
| Cube | 99.42 (2.00) | 99.96 (0.00) | <0.001^a^ | 0.1344 (0.10) | 0.2811 (0.23) | <0.001^a^ |

^a^Mann−Whitney test.

**Supplemental data 2. Analysis of the effects of combinations of subtasks on movement disorders**

| Task Combination | AUC (SE) | *p* value | 95% CI |
| --- | --- | --- | --- |
| Total | 0.9817 (0.0043) | <0.0001 | 0.9732 - 0.9901 |
| L + E | 0.9810 (0.0040) | <0.0001 | 0.9731 - 0.9889 |
| L + E + K + Cu | 0.9808 (0.0039) | <0.0001 | 0.9733 - 0.9884 |
| L + Ch + E + Cu | 0.9796 (0.0044) | <0.0001 | 0.9711 - 0.9881 |
| Ch + E + Cu | 0.9748 (0.0048) | <0.0001 | 0.9654 - 0.9842 |
| E + Cu | 0.9745 (0.0070) | <0.0001 | 0.9608 - 0.9881 |
| L + E + Cu | 0.9745 (0.0047) | <0.0001 | 0.9652 - 0.9837 |
| L + Ch + E | 0.9741 (0.0031) | <0.0001 | 0.9680 - 0.9803 |
| L + E + K | 0.9724 (0.0041) | <0.0001 | 0.9644 - 0.9804 |
| E + K + Cu | 0.9722 (0.0065) | <0.0001 | 0.9594 - 0.9851 |
| Ch + E + K + Cu | 0.9706 (0.0063) | <0.0001 | 0.9583 - 0.9829 |
| L + Ch + E + K | 0.9688 (0.0043) | <0.0001 | 0.9604 - 0.9772 |
| Ch + K + Cu | 0.9580 (0.0159) | <0.0001 | 0.9268 - 0.9892 |
| L + Ch + K + Cu | 0.9578 (0.0104) | <0.0001 | 0.9374 - 0.9783 |
| K + Cu | 0.9487 (0.0168) | <0.0001 | 0.9158 - 0.9816 |
| L + Ch + K | 0.9481 (0.0066) | <0.0001 | 0.9351 - 0.9611 |
| E | 0.9465 (0.0043) | <0.0001 | 0.9381 - 0.9548 |
| Ch + Cu | 0.9437 (0.0197) | <0.0001 | 0.9052 - 0.9822 |
| Ch + E | 0.9407 (0.0038) | <0.0001 | 0.9332 - 0.9482 |
| L + K + Cu | 0.9339 (0.0131) | <0.0001 | 0.9081 - 0.9596 |
| L + K | 0.9270 (0.0093) | <0.0001 | 0.9088 - 0.9452 |
| Ch + E + K | 0.9268 (0.0048) | <0.0001 | 0.9173 - 0.9363 |
| E + K | 0.9225 (0.0046) | <0.0001 | 0.9134 - 0.9316 |
| L + Ch + Cu | 0.9098 (0.0118) | <0.0001 | 0.8867 - 0.9329 |
| L + Ch | 0.9040 (0.0084) | <0.0001 | 0.8876 - 0.9203 |
| Ch | 0.8949 (0.0057) | <0.0001 | 0.8838 - 0.9060 |
| Ch + K | 0.8940 (0.0105) | <0.0001 | 0.8733 - 0.9146 |
| K | 0.8635 (0.0117) | <0.0001 | 0.8406 - 0.8865 |
| Cu | 0.8220 (0.0212) | <0.0001 | 0.7805 - 0.8635 |
| L + Cu | 0.7594 (0.0121) | <0.0001 | 0.7358 - 0.7830 |
| L | 0.6256 (0.0547) | 0.0216 | 0.5184 - 0.7328 |

AUC, area under the curve; L, line; Ch, Chinese; E, English; Cu, cube; K, Korean; SE, standard error; 95% CI, 95% confidence interval.

**Supplemental data 3. Movement disorder subgroup analysis of the effect of language type on Parkinson’s disease**

| Variable | Percentile score | | | Median Weight | | |
| --- | --- | --- | --- | --- | --- | --- |
|  | PD (n=120) | PDS (n=77) | *p* value | PD (n=120) | PDS (n=77) | *p* value |
| Line | 49.29 (56.00) | 68.61 (60.00) | 0.009^a^ | 0.2098 (0.20) | 0.2465 (0.26) | 0.718^a^ |
| Chinese | 17.85 (24.00) | 20.01(31.00) | 0.199^a^ | 0.1942 (0.07) | 0.1933 (0.08) | 0.407^a^ |
| English | 30.64 (31.00) | 49.33 (33.00) | <0.001^a^ | 0.2089 (0.05) | 0.2052 (0.05) | 0.278^a^ |
| Korean | 42.10 (38.00) | 50.64 (41.00) | 0.003^a^ | 0.2064 (0.06) | 0.2122 (0.05) | 0.832^a^ |
| Cube | 42.30 (59.00) | 48.75 (61.00) | 0.154^a^ | 0.1227 (0.07) | 0.1135 (0.08) | 0.544^a^ |

^a^Mann−Whitney test.

**Supplemental data 4. AV133 PET subgroup analysis of the effect of language type on Parkinson’s disease**

| Variable | Percentile score | | | Median Weight | | |
| --- | --- | --- | --- | --- | --- | --- |
|  | PD (n=31) | PDS (n=24) | *p* value | PD (n=31) | PDS (n=24) | *p* value |
| Line | 40.47 (49.00) | 76.96 (50.00) | 0.005^a^ | 0.1921 (0.20) | 0.2341 (0.31) | 0.575^a^ |
| Chinese | 19.60 (32.00) | 17.21 (24.00) | 0.203^a^ | 0.1941 (0.03) | 0.2014 (0.10) | 0.919^a^ |
| English | 33.40 (30.00) | 42.73 (53.00) | 0.067^a^ | 0.2066 (0.03) | 0.2019 (0.07) | 0.879^a^ |
| Korean | 48.82 (36.00) | 48.93 (51.00) | 0.256^a^ | 0.2147 (0.05) | 0.2106 (0.05) | 0.959^a^ |
| Cube | 40.21 (36.00) | 50.33 (67.00) | 0.078^a^ | 0.1514 (0.13) | 0.1002 (0.05) | 0.003^a^ |

^a^Mann−Whitney test.

**Supplemental data 5. Combination analysis of the impact of subtasks on Parkinson’s disease**

| Task Combination | AUC (SE) | *p* value | 95% CI |
| --- | --- | --- | --- |
| L + E + K | 0.7268 (0.0467) | <0.0001 | 0.6352 - 0.8184 |
| L + E + K + Cu | 0.7167 (0.0449) | <0.0001 | 0.6288 - 0.8046 |
| Total | 0.7159 (0.0428) | <0.0001 | 0.6320 - 0.7998 |
| L + Ch + E + K | 0.7130 (0.0422) | <0.0001 | 0.6303 - 0.7957 |
| L + Ch + E + Cu | 0.7010 (0.0381) | <0.0001 | 0.6262 - 0.7757 |
| E | 0.7002 (0.0548) | 0.0003 | 0.5928 - 0.8076 |
| L + E | 0.6983 (0.0453) | <0.0001 | 0.6094 - 0.7871 |
| L + E + Cu | 0.6965 (0.0462) | <0.0001 | 0.6060 - 0.7870 |
| L + Ch + E | 0.6880 (0.0409) | <0.0001 | 0.6078 - 0.7682 |
| E + K + Cu | 0.6879 (0.0514) | 0.0003 | 0.5872 - 0.7886 |
| E + Cu | 0.6855 (0.0573) | 0.0012 | 0.5732 - 0.7978 |
| E + K | 0.6835 (0.0568) | 0.0012 | 0.5722 - 0.7949 |
| Ch + E + K + Cu | 0.6828 (0.0436) | <0.0001 | 0.5973 - 0.7683 |
| L + Ch + K + Cu | 0.6827 (0.0257) | <0.0001 | 0.6322 - 0.7331 |
| L + K | 0.6795 (0.0352) | <0.0001 | 0.6105 - 0.7486 |
| L + Ch + K | 0.6710 (0.0280) | <0.0001 | 0.6162 - 0.7258 |
| L + K + Cu | 0.6649 (0.0350) | <0.0001 | 0.5963 - 0.7336 |
| Ch + E + Cu | 0.6617 (0.0392) | <0.0001 | 0.5849 - 0.7384 |
| Ch + E + K | 0.6589 (0.0433) | 0.0002 | 0.5740 - 0.7437 |
| L + Ch + Cu | 0.6475 (0.0182) | <0.0001 | 0.6118 - 0.6833 |
| Ch + E | 0.6459 (0.0398) | 0.0002 | 0.5678 - 0.7240 |
| Ch + K + Cu | 0.6348 (0.0357) | 0.0002 | 0.5648 - 0.7049 |
| K + Cu | 0.6304 (0.0553) | 0.0184 | 0.5220 - 0.7388 |
| K | 0.6258 (0.0354) | 0.0004 | 0.5563 - 0.6952 |
| L + Cu | 0.6203 (0.0219) | <0.0001 | 0.5774 - 0.6633 |
| L + Ch | 0.6193 (0.0204) | <0.0001 | 0.5792 - 0.6593 |
| Ch + K | 0.6156 (0.0311) | 0.0002 | 0.5545 - 0.6766 |
| L | 0.6103 (0.0167) | <0.0001 | 0.5776 - 0.6430 |
| Ch + Cu | 0.5987 (0.0345) | 0.0042 | 0.5311 - 0.6663 |
| Cu | 0.5603 (0.0699) | 0.3888 | 0.4232 - 0.6974 |
| Ch | 0.5543 (0.0138) | <0.0001 | 0.5272 - 0.5814 |

AUC, area under the curve; L, line; Ch, Chinese; E, English; Cu, cube; K, Korean; SE, standard error; 95% CI, 95% confidence interval.
