# Supplementary material for: Multitype hand writing as a digital marker for Parkinson's disease
Source: Clin Park Relat Disord. 2026 Jul 2;15:100480. doi: 10.1016/j.prdoa.2026.100480 (PMC13377146; doi:10.1016/j.prdoa.2026.100480)
Supplement: Appendix B — Supplementary material 2 [file mmc2.docx]

**Supplementary Methods: Detailed Data Processing and Feature Engineering Pipeline**

This document provides a comprehensive description of the data processing and feature engineering pipeline used to convert raw time-series signals from the Wacom tablet into two distinct structured datasets: (1) a set of multi-channel time-series data for deep learning analysis, and (2) a large-scale summary feature pool for statistical biomarker analysis.

1. **Signal Preprocessing**

Prior to feature extraction, the raw data for each participant underwent several preprocessing steps:

1. Unit Conversion: The raw x-y coordinates, originally recorded in device-specific units, were converted to centimeters (cm) by applying a scaling factor of 1/1000.
2. Timestamp Normalization: The original timestamps were converted to seconds. For each recording, the timeline was normalized to start at t=0 by subtracting the timestamp of the first data point.
3. Event Type Encoding: The categorical Event Type data (e.g., 'pen Down', 'pen Dragged', 'pen Up', 'pen Moved') was mapped to integer values (1, 2, 3, 4, respectively) for computational analysis.
4. Anomaly Removal: Data points where both x and y coordinates were simultaneously zero after the initial point were considered anomalous and removed from the time series.
5. **Generation of Time-Series Features**

From the preprocessed data, a set of 24 dynamic time-series signals was engineered for each participant. These signals form the basis for both the deep learning models and the subsequent statistical feature pool construction. They are categorized as follows:

**2.1. Instantaneous Kinematic and Dynamic Characteristics (IKDC)**

For each time point t_i_, a comprehensive set of instantaneous features was calculated based on the preprocessed signals. Let (x_i_,y_i_) be the coordinates, p_i_ be the pressure, and Δt_i_=t_i_−t_i−1_ be the time interval from the previous point.

1. **Kinematic Derivatives**

The primary kinematic parameters—velocity (v), acceleration (a), and jerk (j)—were computed using first-order finite differences.

**Velocity:** The horizontal (v_x,i_) and vertical (v_y,i_) components were calculated as:

v_x,i_=x_i_−x_i−1_/Δt_i_; v_y,i_=yi−y_i−1_/Δti​​

**Acceleration:** The horizontal (a_x,i_) and vertical (a_y,i_) components were calculated as:

a_x,i_=v_x,i_−v_x,i−1_/Δt_i_; a_y,i_=v_y,i_−v_y,i−1_/Δti​​

**Jerk:** The horizontal (jx,i) and vertical (jy,i) components were calculated as:

j_x,i_=a_x,i_−a_x,i−1_/Δt_i_; j_y,i_=a_y,i_−a_y,i−1_/Δt_i_​​

To handle outliers and signal artifacts, two correction steps were applied. First, for any time interval Δt_i_>1.0 s (indicating a signal dropout), all kinematic values at that point were set to zero. Second, acceleration and jerk values were clipped to predefined thresholds (±2000.0 cm/s² for acceleration and ±2,000,000.0 cm/s³ for jerk) to mitigate the impact of noise.

1. **Composite, Geometric, and Dynamic Parameters**

Additional parameters were derived to provide a richer description of the pen-tip dynamics:

**Total Velocity & Acceleration:** Calculated as the Euclidean norm of their respective components:

v_total,i_=(v_x,i_^2^+v_y,i_^2^)^1/2^ ; a_total,i_=(a_x,i_^2^+a_y,i_^2^)^1/2^​

**Displacement increment & Cumulative Displacement:** The displacement increment (delta distance) Δdi​ was calculated as the Euclidean distance between consecutive points. The cumulative displacement at point i is the sum of all preceding increments:

Δd_i_=[(x_i_−x_i−1_)^2^+(y_i_−y_i−1_)^2^]^1/2^ ; Di=$\sum_{k=1}^{i} \Delta d_{k}$

**Motion Direction Angle:** The angle of the pen-tip movement vector at each time point, calculated in degrees using arctan2(dy, dx).

**Pressure Change Rate:** The rate of change of pen pressure, calculated as:

p_i_′=p_i_−p_i−1_/Δt_i​_​

**Trajectory Curvature (κ):** The curvature of the trajectory at point i was calculated using the formula for a parametric curve:

$$\kappa_{i}=\frac{\left| v_{x,i}a_{y,i}-v_{y,i}a_{x,i} \right|}{\left( {v^{2}}_{x,i}+{v^{2}}_{y,i} \right)^{\frac{3}{2}}}$$

**2.2. Calculation of Behavioral Segmentation Characteristics (BSC)**

The BSC features provide macroscopic descriptions of distinct behavioral phases. The Event Type signal was used to parse each writing sequence into two fundamental stages:

1. ‘Strokes’ Stage: Segments where the pen tip is in contact with the tablet surface (i.e., pressure > 0), typically initiated by a 'pen Down' event and concluded by a 'pen Up' event.
2. ‘Flights’ Stage: Segments where the pen tip is moving in the air above the tablet surface (i.e., pressure = 0), typically occurring between two consecutive strokes.

For each independent stroke and flight segment, the following macroscopic features were calculated:

1. Duration: The total time elapsed from the beginning to the end of the segment, measured in seconds.
2. Total Trajectory Length: The sum of all displacement increments (Δd_i_) within the segment.
3. Horizontal and Vertical Amplitudes: The range of motion within the segment, defined as the difference between the maximum and minimum coordinates on each axis (width=x_max_−x_min_; height=y_max_−y_min_)
4. **Construction of the Large-Scale Derived Feature Pool**

For the statistical biomarker analysis (PD vs. PDS group comparison), the 24 time-series signals generated in the previous step were used to construct a high-dimensional summary feature pool. This was achieved by applying the calculate_advanced_stats_for_signal function to each of the 24 signals for every participant's writing task. This function calculates a comprehensive set of statistical descriptors for each signal, including:

1. Metrics of Central Tendency and Dispersion: Mean, standard deviation, median.
2. Robust Statistical Metrics: 25th (p25) and 75th (p75) percentiles, interquartile range (iqr), 10% trimmed mean (trimmed_mean_10%), and 5th (p05) and 95th (p95) percentiles as robust alternatives to minimum and maximum.
3. Metrics of Distribution Shape: Skewness (skew) and kurtosis (kurt).
4. Time-Domain Metrics: Root mean square (rms), reflecting signal energy.
5. Frequency-Domain Metrics: Derived from a Fast Fourier Transform (FFT), this includes the dominant frequency (fft_dominant_freq) and the energy ratio within the 4-6 Hz tremor band (fft_tremor_ratio).
6. Non-linear Dynamics Metrics: Sample Entropy (sampen), calculated using the nolds library, to quantify the complexity and regularity of the signal.

This process resulted in a final feature matrix where each row represents a participant and each column represents a specific statistical summary of one of the 24 original time-series signals.
